# Diabetic foot risk factors in type 2 diabetes patients: a cross-sectional case control study

**DOI:** 10.1186/2251-6581-13-79

**Published:** 2014-08-04

**Authors:** Piotr Nehring, Beata Mrozikiewicz-Rakowska, Monika Krzyżewska, Agnieszka Sobczyk-Kopcioł, Rafał Płoski, Grażyna Broda, Waldemar Karnafel

**Affiliations:** 1 Gastroenterology and Metabolic Diseases Department, Medical University of Warsaw, ul. Banacha 1a, Warsaw 02-097, Poland; 2 Department of General Biology and Parazytology, Medical University of Warsaw, Warsaw, Poland; 3 Department of Medical Genetic, Medical University of Warsaw, Warsaw, Poland; 4 Department of Epidemiology, Cardiovascular Disease Prevention and Health Promotion, Institute of Cardiology in Warsaw, Warsaw, Poland

**Keywords:** Diabetes, Diabetic foot, Epidemiology, Type 2 diabetes

## Abstract

**Background:**

Diabetic foot is a serious condition in patients with a long lasting diabetes mellitus. Diabetic foot treated improperly may lead not only to delayed ulceration healing, generalized inflammation, unnecessary surgical intervention, but also to the lower limb amputation. The aim of this study was to compare diabetic foot risk factors in population with type 2 diabetes and risk factors for diabetes in healthy subjects.

**Methods:**

The study included 900 subjects: 145 with diabetic foot, 293 with type 2 diabetes without diabetic foot and 462 healthy controls matched in terms of mean age, gender structure and cardiovascular diseases absence. Study was conducted in Gastroenterology and Metabolic Diseases Department, Medical University of Warsaw, Poland. In statistical analysis a logistic regression model, *U* Mann-Whitney’s and *t*-Student test were used.

**Results:**

The binomial logit models analysis showed that the risk of diabetic foot in patients with type 2 diabetes was decreased by patient’s age (odds ratio [OR] = 0.94; 95% confidence interval [CI]: 0.92-0.96; p = 0.00001) and hyperlipidaemia (OR = 0.54; 95% CI: 0.36-0.81; p = 0.01). In contrast, male gender (OR = 2.83; 95% CI: 1.86-4.28; p = 0.00001) diabetes duration (OR = 1.04; 95% CI: 1.03-1.06; p = 0.0003), weight (OR = 1.04; 95% CI: 1.03-1.06; p = 0.00001), height (OR = 1.08; 95% CI: 1.05-1.11; p = 0.00001) and waist circumference (OR = 1.028; 95% CI: 1.007-1.050; p = 0.006) increase the risk of diabetic foot. The onset of type 2 diabetes in healthy subjects was increased by weight (OR = 1.035; 95% CI: 1.024-1.046; p = 0.00001), WC (OR = 1.075; 95% CI: 1.055-1.096; p = 00001), hip circumference (OR = 1.03; 95% CI: 1.01-1.05; p = 0.005), overweight defined with body mass index (BMI) above 24,9 kg/m^2^ (OR = 2.49; 95% CI: 1.77-3.51; p = 0.00001) and hyperlipidaemia (OR = 3.53; 95% CI: 2.57-4.84; p = 0.00001).

**Conclusions:**

Risk factors for Type 2 diabetes and diabetic foot are only partially common. Study proved that patients who are prone to developing diabetic foot experience different risk factors than patients who are at risk of diabetes. Identification of relationship between diabetic foot and diabetes risk factors in appropriate groups may help clinicians to focus on certain factors in diabetic foot prevention.

## Introduction

The diabetic clinics’ registers data shows that type 2 diabetes is present in 5.37% of adult population, however detail epidemiological studies conducted in several regions of Poland suggest twice as high frequency [1]. It means, that very similar number of individuals is unaware of having diabetes. As a result, there is a large number of patients in whom diagnosis is made when diabetes complications (e.g. diabetes foot) are already present. According to Abouaesh et al. diabetic foot is present in 15% of general population with diabetes and it is estimated that it affects 2-12.1% of patients with type 2 diabetes [2,3].

Diabetic foot is a long lasting diabetes complication developing upon the presence of neuropathy and peripheral arterial disease (PAD) as basic etiological factors [4]. The sensorial nerve fibers damage results with the impaired sensation of pain, temperature and vibration leading to frequent, firstly unnoticed foot injuries with a callus and wound as a consequence. Motor neuropathy results in loss of foot muscles function, that leads to deformation in foot shape and increases the risk of injury. The characteristic triad: neuropathy, deformation and injury is present in 60% of patients [5]. The recurrence ratio is very high, affecting 25-80% of patients with type 2 diabetes per year [6].

Several studies showed 15-46 fold higher lower limb amputation risk (LLA) in patients with diabetes in comparison with general population. Moreover, the neuropathy as a consequence of long lasting diabetes, is a cause of 50-70% of non-traumatic LLA [3,7]. According to Mayfield et al. studies, the odds of LLA in patients with three or four risk factors (neuropathy, PAD, foot bones deformation and foot wound history) are 9 times higher comparing to patients with only one risk factor [8]. Moreover, an amputation increases the risk of subsequent LLA and mortality of patients. Mortality within the first month after LLA is 8.5% of patients, and in 5 year period reaches 39-68% [9]. Furthermore, the diabetic foot treatment expenses absorb about 15% of overall hospital budget for diabetes [3].

The aim of this study was to compare diabetic foot risk factors in population with type 2 diabetes and risk factors of diabetes in healthy subjects.

## Methods

The study was conducted in Gastroenterology and Metabolic Diseases Department, Medical University of Warsaw, Poland. The study included 900 individuals: 145 patients with diabetic foot, 293 with type 2 diabetes without diabetic foot and 462 healthy control subjects. The control group consisted of randomly selected individuals, matched with age, gender structure and lack of cardiovascular conditions. The proportion of healthy controls per one patient with diabetic foot was 3.19:1. All patients underwent an examination with a survey designed by authors. Diabetic foot was diagnosed according to Global consensus guidelines on the management and prevention of the Diabetic Foot criteria, as an a wound, infection and/or deep foot tissues destruction localized in lower limb below the ankle in patients with diabetes complicated with neuropathy and/or PAD [10].

The diabetic foot type was defined with detailed physical examination of superficial sensation impairment. Neuropathy was evaluated using Thermo-tip (temperature), monofilament (touch), Neuro-tip (pain) and Semmens-Weinstein pitchfork (vibration). The presence of pulse was assessed on dorsal pedis and tibial posterior arteries. Each patient was qualified according to ankle-brachial index (ratio of the blood pressure in the lower legs to the blood pressure in the arms assessed with a typical sphygmomanometer and a blind Doppler, Bidop ES-100 V3, Hadeco Inc., Kawaski, Japan.) and referred to ultrasound Doppler or to a vascular surgeon consultation if necessary. When a painless ulceration was present, a diabetic foot of neuropathic origin was diagnosed. The criteria of hyperlipidaemia were hipercholesterolaemia, hipertrigliceridaemia or lipid-lowering medications intake. Individuals with dominating angiopathic etiology of diabetic foot were disqualified from the study. The statistical analysis was performed with the use of logistic regression models, *U* Mann–Whitney and t-*S*tudent tests with STATISTICA 9PL (StatSoft, Inc.) software. The anthropometric features of type 2 diabetes patients and healthy control groups were previously presented in the article on the same population in Polish Archives of Internal Medicine [11]. Missing data were deleted in pair wise. Missing data were deleted pairwise.

## Results

### Diabetic foot risk factors in type 2 diabetes patients

The characteristics of the study groups are presented in Table 1. The logistic regression analysis showed that protecting factor against diabetic foot occurrence in type 2 diabetic population was patients age and hyperlipidaemia (mean 62.97 *v* 71.27 years, OR = 0.94, 95% CI: 0.92-0.96, p = 0.00001 and OR = 0.54, 95% CI: 0.36-0.81, p = 0.01 respectively). (Table 2).

**Table 1:** Characteristics of the studied group

		**Diabetic foot**	**Missing data n/N**	**Diabetes mellitus type 2 [**[11]**]**	**Missing data n/N**	**Control group [**[11]**]**	**Missing data n/N**
Total number		145	0/145	293	0/293	462	0/462
Female/male, %		34/66	0/49; 0/96	59/41	0/173; 0/120	52/48	0/239; 0/223
Mean age, y ± SD							
	Male	72.42 ± 10.46	3/96	57.35 ± 6.45	4/120	62.11 ± 10.63	0/223
	Female	69.65 ± 11.53	2/49	58.87 ± 6.78	10/173	64.92 ± 11.95	0/293
Mean diabetes duration, y ± SD		15.79 ± 9.99	4/145	12.28 ± 8.95	15/293	N/A	N/A
Mean weight, kg ± SD		93.09 ± 17.40	18/145	80.72 ± 16.82	12/293	73.08 ± 13.44	0/462
Mean height, cm ± SD		171.92 ± 8.58	25/145	165.48 ± 9.32	8/293	165.69 ± 8.62	0/462
Mean body mass index, kg/m^2^ ± SD		32.36 ± 5.35	7/145	29.93 ± 5.83	15/293	26.58 ± 4.20	1/462
Mean waist circumference, cm ± SD		111.10 ± 13.89	65/145	104.55 ± 17.25	197/293	91.62 ± 11.81	4/462
Mean hip circumference, cm ± SD		110.92 ± 12.07	64/145	108.47 ± 12.74	197/293	105.00 ± 9.72	20/462

**Table 2:** Factors decreasing the risk of diabetic foot in type 2 diabetes population (p < 0.05)

**Studied groups**	**Diabetic foot**	**Missing data n/N**	**Type 2 diabetes without diabetic foot**	**Missing data n/N**
Mean age, y	62.97 ± 11.12	5/145	71.27 ± 11.00	14/293
Hyperlipidaemia, %	40.43 (57/141)	4/145	55.88 (152/272)	21/293

**Figure 1:**
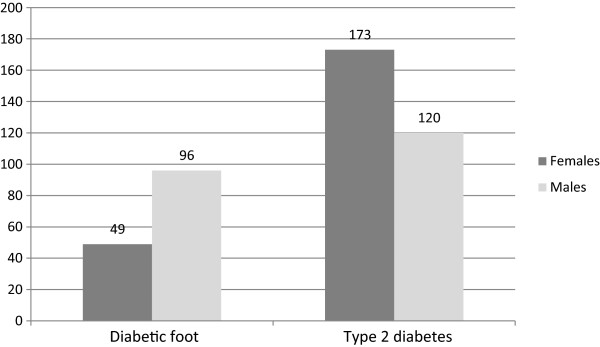
The gender structure of patients with diabetic foot and type 2 diabetes group.

The presence of diabetic foot was increased by male gender (OR = 2.83, 95% CI: 1.86-4.28, p = 0.00001), duration of diabetes (mean 15.83 *v* 12.27 years, OR = 1.04, 95% CI: 1.03-1.06, p = 0.0003), weight (mean 93.01 *v* 80.72 kg, OR = 1.04, 95% CI: 1.03-1.06, p = 0.00001), height (mean 172.01 *v* 165.48 cm, OR = 1.08, 95% CI: 1.05-1.11, p = 0.00001) and WC (mean 111.15 *v* 104.55 cm, OR = 1.028, 95% CI: 1.007-1.050, p = 0.006) (Figure 1).

### Risk factors for type 2 diabetes in a general population

**Figure 2:**
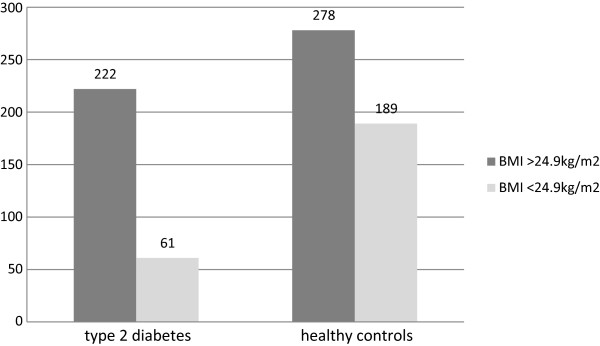
**Number of BMI over and below 24.9 kg/m**^**2 **^**in patients with type 2 diabetes and in healthy controls.**

In the presented study, the comparison between type 2 diabetes group and healthy subjects showed that factors increasing the risk of diabetes were weight (mean 80.72 *v* 73.09 kg, OR = 1.035, 95% CI: 1.024-1.046, p = 0.00001), WC (mean 104.55 *v* 91.62 cm, OR = 1.075, 95% CI: 1.055-1.096, p = 0.00001), hips circumference (mean 108.47 *v* 105.00 cm, OR = 1.03, 95% CI: 1.01-1.05, p = 0.005), overweight defined as BMI over 24.9 kg/m^2^ (OR = 2.49, 95% CI: 1.77-3.51, p = 0.00001) (Figure 2).

In addition, the presence of hyperlipidaemia increased the risk of type 2 diabetes in comparison to healthy controls (OR = 3.53, 95% CI: 2.57-4.84, p = 0.00001) (Table 3).

**Table 3:** Risk factors of type 2 diabetes in general population (p < 0.05)

	**Type 2 diabetes without diabetic foot**	**Missing data n/N**	**Healthy controls**	**Missing data n/N**
Mean weight, kg	80.72 ± 16.82	12/293	73.08 ± 13.44	0/462
Mean waist circumference, cm	104.55 ± 17.25	197/293	91.62 ± 11.81	4/462
Mean hips circumference, cm	108.47 ± 12.74	197/293	105.00 ± 9.72	20/462
Mean BMI > 24.9 kg/m^2^, %	79.00 (222/281)	12/293	60.22 (278/467)	0/462
Hyperlipidaemia, %, (n/N)	55.88 (152/272)	21/293	26.35 (122/462)	0/462

## Discussion

The outcome of this study contributes to developement of knowledge about the risk factors for diabetic foot in Polish population with type 2 diabetes. This findings may be helpful in clinical practice to identify individuals prone to development of diabetic foot. Our study showed that tall men with type 2 diabetes are at high risk of diabetic foot. The increased incidence of diabetes complications in men was also presented in the study conducted by Simon et al., who indicated 1.4-fold increased prevalence of diabetes complications among men comparing to woman [12,13]. Our results concerning the risk of diabetic foot associated with patient’s height are similar to findings described by Sosenko et al. [14]. This effect is probably related to increased risk of demyelination process in tall patients, comparing to individuals with shorter lower limbs nerve fibers. The correlation of diabetes duration and diabetic foot risk is corresponding with studies performed by several authors, e.g. Ashok et al. [14-20]. According to Al-Maskari's studies the risk of neuropathy is increased after 10-12 years of type 2 diabetes duration [13].

Very important finding in our study is the existence of factors commonly and irrespectively increasing the risk of diabetic foot and type 2 diabetes. Only patient’s body weight and waist circumference length increases both diabetic foot and type 2 diabetes occurrence risk. Presented results are in correspondence with Boyko et al. studies, who described that the risk of diabetic foot increases when patient's weight is 20 kg higher than optimal body weight [4].

The results of this study demonstrates that waist and hip circumference, overweight and hyperlipidaemia increases the odds of type 2 diabetes in general population what is in correspondence with other studies [21,22]. Dehghan et al., demonstrates that important diabetes risk factors are BMI (over 25 kg/m^2^) and hip circumference (over 102 cm in men and over 88 cm in women) [21]. Haffner et al. indicates that type 2 diabetes risk factors are age, male sex, BMI (over 27.7 kg/m^2^), waist-hip ratio over 0.825 in women and over 0.938 in men [22]. Other studies show correlation between metabolic control of diabetes and the incidence of neuropathy, diabetic foot and even LLA [14,16-18,20,23].

Worthy notice are Booy et al. studies, showing no correlation between the risk of neuropathy in type 2 diabetes patients and the frequency of hyperlipidaemia, hypertension or smoking [24]. The presented study confirms no correlation with higher incidence of diabetic foot in population with hyperlipidaemia.

The strength of our study is a consistence of two separate control groups (individuals with uncomplicated type 2 diabetes and healthy controls). Strict controls matching with cases due to anthropometric and clinical features was very important. The limitation of this study is lack of division on neuropathic and mixed type of diabetic foot, however, individuals with dominating ischemic etiology were excluded.

There is a need to investigate the subject of diabetic foot risk factors in more numerous study groups and to extend it on other populations.

## Conclusions

It is possible to indicate common factors influencing diabetic foot and diabetes frequency that are part of the etiopathogenetic process. There are also factors that specifically increase risk of either type 2 diabetes or diabetic foot after the occurrence of diabetes. The study also showed the existence of independent prognostic factors for diabetic foot that are unrelated to type 2 diabetes risk factors of high importance in population with actually developed diabetes.

## Abbreviations

OR: Odds ratio; CI: Confidence interval; WC: Waist circumference; BMI: Body mass index; PAD: Peripheral arterial disease; LLA: Lower limb amputation.

## Competing interest

The author(s) declare that they have no competing interests.

## Authors’ contribution

The study comprised: study design, data collection, scientific analysis, statistical analysis and manuscript preparation. PN was responsible for data collection, statistical analysis and manuscript preparation. BM-R was responsible for study design, data collection, scientific analysis and manuscript preparation. MK was collecting data and preparing manuscript. AS-K was designing study, granted statistical support and prepared manuscript. RP was responsible for study design and statistical analysis. GB designed study and collected data. WK was granting scientific support and collecting data. All authors read and approved the final manuscript.
